# Research on and Model Analysis of Flexural Mechanical Properties of Basic Magnesium Sulfate Cement Concrete Beams

**DOI:** 10.3390/ma17081761

**Published:** 2024-04-11

**Authors:** Qiquan Mei, Yuning Gao, Hongfa Yu, Haiyan Ma, Xiangchao Zeng, Lingyu Li, Jianbo Guo

**Affiliations:** 1 School of Civil Engineering, San Jiang University, Nanjing 210012, China; meiqiquan@nuaa.edu.cn; 2 College of Civil Aviation, Nanjing University of Aeronautics and Astronautics, Nanjing 211106, China; mahaiyan@nuaa.edu.cn (H.M.); tx0335@163.com (X.Z.); lilingyu990322@163.com (L.L.); guojianbo@nuaa.edu.cn (J.G.); 3 School of Civil and Architectural Engineering, Yangtze Normal University, Chongqing 408100, China

**Keywords:** Basic Magnesium Sulfate Cement Concrete (BMSC), Ordinary Portland Cement Concrete (OPC), cracking moment, ultimate bending moment, beam

## Abstract

This study presents a comprehensive investigation into the mechanical properties of Basic Magnesium Sulfate Cement Concrete (BMSC) in comparison to Ordinary Portland Cement Concrete (OPC) within reinforced concrete components. The main objective is to evaluate BMSC’s applicability for practical engineering purposes, with a focus on its with early high strength, improved toughness, and superior crack resistance compared to conventional concrete. Experimental procedures involved fabricating beam specimens using OPC concrete with a C40 strength grade, alongside BMSC beams with varying strength grades (C30, C40, and C50). These specimens underwent bending resistance tests to analyze crack patterns and mechanical characteristics. The findings reveal that BMSC beams demonstrate enhanced bending and tensile properties at equivalent strength grades compared to OPC beams. Particularly, cracking mainly occurred at the mid-span region of BMSC beams, characterized by narrower cracks, indicating superior crack resistance. However, it was noted that the toughness of BMSC beams decreases as the strength grade increases. The maximum mid-span deflection of the BMSC test beam was smaller than that of the OPC test beam, which was 3.8 mm and 2.6 mm, respectively. The maximum crack width of the OPC beam was 4.7 times that of the BMSC beam. To facilitate practical implementation, the study developed calculation models for estimating the crack bending distance and ultimate bending distance in BMSC beams, offering valuable tools for engineering design and optimization. Overall, this research provides significant insights into the mechanical behavior of BMSC, presenting potential advantages for structural engineering applications.

## 1. Introduction

### 1.1. Research Status

Reinforced concrete structures combine the advantages of both steel and concrete. Their low cost makes them a preferred choice for civil engineering structural designs. These structures have a wide range of applications and have remained one of the most important engineering structural forms in the 21st century. Among the various materials used in concrete structures, Ordinary Portland Cement (OPC) Concrete is particularly significant. It offers numerous advantages, such as abundant raw materials, ease of construction, controllable strength, high compressive strength, and good durability. However, it also has some drawbacks, including significant self-weight, low tensile strength, and poor crack resistance [1,2,3].

Generally, methods to enhance the tensile strength of concrete in engineering projects include the addition of fiber-reinforced materials, increasing the strength grade of the concrete, and using cementing materials with high tensile strength [4,5]. Although incorporating steel fiber or enhancing the strength grade are common approaches to improve concrete’s tensile strength, adding steel fiber significantly increases the cost, making its widespread application in large-scale projects difficult. Moreover, indiscriminately increasing the concrete’s strength grade can result in the unnecessary use of cementing materials and an increase in the concrete’s brittleness, which is not desirable. Developing a cementing material with high tensile strength may be an ideal solution to achieve high tensile strength in cement concrete.

After years of research and exploration, Yu Hongfa et al. [6] developed a new type of magnesium cementing material: Basic Magnesium Sulfate Cement (BMSC), part of the MgO-MgSO_4_-H_2_O cementing material system. Its main hydration product is a newly discovered basic magnesium sulfate whisker phase with the chemical formula 5Mg(OH)_2_·MgSO_4_·7H_2_O, belonging to the monoclinic crystal system and the C121 space group. The crystal structure features a layered composition built from an MgO_6_ octahedral skeleton. BMS offers advantages in mechanical properties, such as with early high strength, enhanced tensile strength, and high bending resistance. Concrete made with this cementing material exhibits exceptional mechanical properties, including extremely high compressive and tensile strengths, a static elastic modulus, and impact toughness. These properties make it suitable for high-rise or super high-rise building components, like beams, columns, and joints, potentially reducing the cross-sectional size of components, improving structural seismic capabilities, and increasing usable building space. BMSC boasts advantages, such as water resistance, rapid setting, early strength, and high strength. Its physical properties are essentially akin to those of ordinary Portland Cement [7].

The potential applications of BMSC concrete in structural engineering are extensive, ranging across fields like bridge and pavement engineering, construction, hydraulic engineering, special structures, and military applications, indicating significant research and development potential [8]. Firstly, it demonstrates higher early and ultimate strengths, leading to quicker strength development during construction and improved construction efficiency [9]. Secondly, BMSC possesses excellent crack resistance, effectively mitigating crack propagation due to temperature variations and load actions, thus enhancing structural durability and safety [10,11]. Additionally, BMSC showcases exceptional resistance to chemical attacks, such as sulfate and chloride ion ingress, prolonging the service life of structures. Compared to OPC concrete of the same strength grade, BMSC concrete has more than twice the tensile strength and boasts benefits, such as high rigidity, rapid setting, early strength, high fire resistance, low thermal conductivity, water resistance, corrosion resistance, and carbonization resistance [9,12]. Ordinary Portland Cement is primarily composed of limestone, clay, and iron ore. Its production process involves the high-temperature calcination of raw materials, consuming substantial energy, thus resulting in significant carbon dioxide emissions. Additionally, the production of Portland Cement generates pollutants, such as dust and wastewater, contributing to environmental pollution in the surrounding areas. On the other hand, magnesium sulfate cement, derived mainly from magnesium oxide and magnesium sulfate, exhibits relatively lower energy consumption during production compared to ordinary Portland Cement. Under specific conditions, the preparation process of magnesium sulfate cement can yield lesser carbon dioxide emissions. Particularly, when employing lithium extraction from salt lakes (Salt Lake lithium extraction technology) to produce magnesium sulfate cement, it facilitates the simultaneous acquisition of lithium resources, thereby reducing carbon emissions [13]. Consequently, BMSC presents vast potential as a new cementitious material.

However, the application and development of BMSC in reinforced concrete are still in their initial stages [13,14,15], primarily focusing on non-load-bearing components, like external wall insulation and interior decoration. Research progress on BMSC abroad indicates significant advancements in material properties, manufacturing processes, and engineering applications. In terms of material properties research, international scholars have extensively investigated BMSC’s mechanical properties, durability, and shrinkage behavior, among other crucial aspects. Chengyou Wu [16] believes that within 50 freeze–thaw cycles, the mass loss and relative dynamic modulus reduction rate of BMSC concrete specimens are significantly lower than that of Portland Cement Concrete. The frost resistance of BMSC concrete is obviously better than that of OPC concrete. Zeng [16] and Yue [17] specifically studied and analyzed the mechanical properties of reinforced BMSC concrete beams, concluding that BMSC beams have a higher bending capacity than OPC beams. Their experimental results indicated that the cracking bending distance of BMSC beams is about 15% greater than that of OPC beams with the same reinforcement ratio and strength grade. Furthermore, the model calculation formula for OPC beams could also be applied to BMSC beams.

The mechanical properties of beams with varying grades may follow different patterns and yield distinct conclusions. To explore the practical application of BMSC concrete in load-bearing structures, particularly in frame constructions, and to gain a detailed understanding of its mechanical properties for broader use in civil engineering, further experimental research on the mechanical properties of components made from BMSC concrete is essential.

### 1.2. Research Significance

The study of concrete beams utilizing sulfate cement holds significant implications for sustainable construction.

Low-Carbon Raw Materials: Magnesium oxide and magnesium sulfate sourced from Qinghai Salt Lake are attractive due to their minimal carbon footprint. Their production processes do not involve direct carbon emissions, contributing to a reduction in the environmental impact of concrete production [18,19].

Potential for Magnesium Cement in Construction: Magnesium cement building materials have already seen some application in the Qinghai area, including structural load-bearing elements. This paves the way for further research and the utilization of magnesium cement as a viable structural material in civil engineering. Potential applications for this new magnesium cement are wide-ranging, including building components for herder settlements, fire boards, ventilation pipes, activity rooms, partition and roof panels, blocks, building decorations, packing boxes, sleepers, support structures, and hot-pressed magnesium cement panels.

Case Study: Tuergan Village Prefabricated Structure: Tuergan Village, a demonstration site for rural revitalization in Qinghai Province and a designated “characteristic town” in China, serves as a successful example of prefabricated Building Material Science and Engineering (BMSC) concrete frame structures. The project boasts a building area of 717.72 sqm (main building area: 621.46 sqm). The structural design employs the “equivalent cast-in-place” method, with a seismic fortification intensity of 7 degrees. The two-story (with local three-story sections) main structure utilizes BMSC concrete with a design grade of C30. Notably, the assembly rate of building components exceeds 50%. Construction involved the on-site precasting of BMSC concrete beams and columns, followed by the pouring of BMSC concrete at fabricated joints. The CL wall panel assembly and wall plastering were completed subsequently. The project achieved impressive results: the 28-day average compressive strength of BMSC cube specimens on-site exceeded the design strength grade by 118% to 180%.

Enhanced Durability and Strategic Importance: The technological breakthrough in sulfur-oxygen magnesium cement in 2013 has led to significant improvements in durability, suggesting a longer service life and superior performance in real-world engineering applications. Furthermore, the abundance of raw materials in western China, coupled with the government’s focus on developing low-carbon concrete, underscores the strategic importance of researching sulfate cement concrete. This aligns perfectly with national environmental goals and low-carbon initiatives in western provinces, offering a promising path for sustainable infrastructure development and economic growth.

## 2. Materials and Methods

### 2.1. The Design and Mix Proportions of the Specimens

#### 2.1.1. Material

The cement used in the test comprised OPC produced by Jiangsu Jinfeng Cement Group Co., Ltd. (Liyang, China), and BMSC produced by Shenyang Jinhui Landing Co., Ltd. (Shenyang, China) with their basic physical properties detailed in Table 1. The main components of BMSC include light-burned MgO (LBM) from Haicheng, Liaoning Province, MgSO_4_·7H_2_O from Zibo, Shandong Province, and a core admixture of citric acid (CA) [18]. Grade II fly ash from the Fushun Thermal Power Plant (Fushun, China) was used, characterized by a fineness of 0.04 mm, a sieve residue rate of 9%, and a water demand ratio of 104. The ground slag, sourced from the Anshan Iron and Steel Slag Development Company (Anshan, China), had a specific surface area of 430 m^2^/kg and a density of 2.80 g/cm³. The chemical compositions of light-burned MgO (LBM), fly ash, and slag are presented in Table 2 [18,20,21]. For the test, gravel from the Xingyuan Mine (Guangde, China) was used as stone, with a maximum particle size of 16 mm, an apparent density of 2610 kg/m³, a loose density of 1440 kg/m³, a needle-like particle content of 4.8% (not exceeding 12%), and a crushing value of 10.4, fitting the continuous gradation range of 5–16 mm. The water used in the test was tap water from Liyang City, meeting national standards.

#### 2.1.2. Specimen Size and Mix Ratio

**Figure 1:**
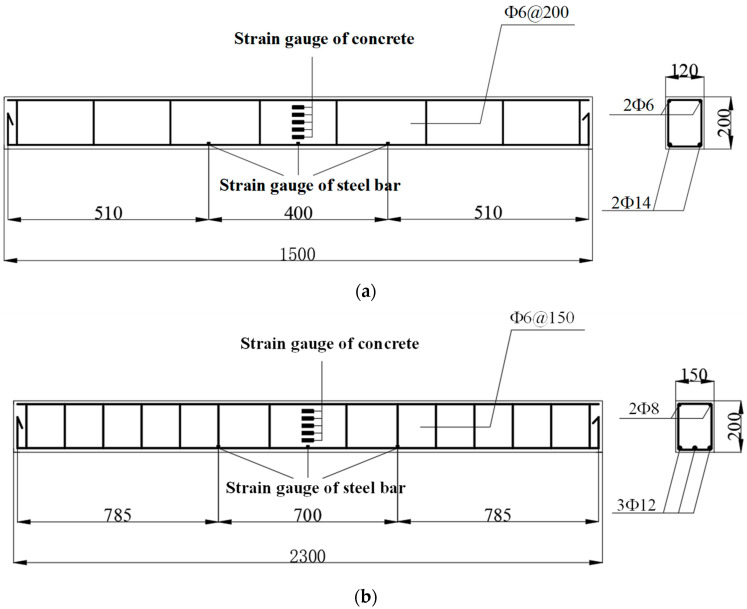
Details of beam size and section reinforcement. (**a**) L = 1500, b × h = 120 mm × 200 mm. (**b**) L = 2300 mm, b × h = 150 mm × 200 mm.

**Figure 2:**
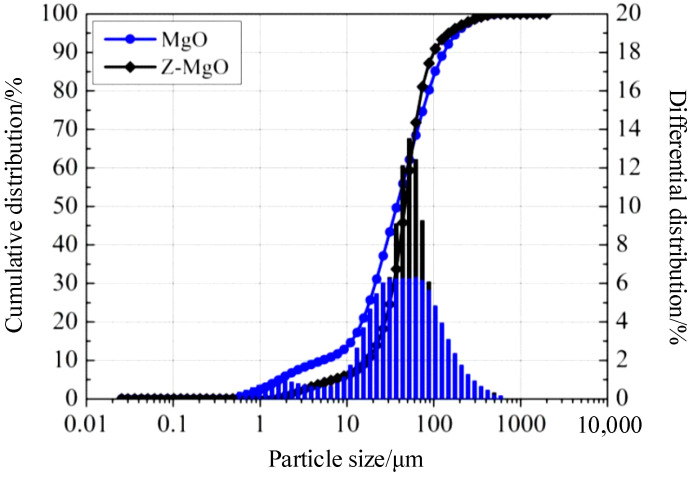
Size distribution curve of magnesium oxide.

In this study, ordinary cement and BMSC were used to design reinforced beams with strength grades of C30, C40, and C50, adjusting the water–binder ratio accordingly. The fit ratio and strength of specimens are shown in Table 3. Two different sizes of specimens were designed, each with a rectangular cross-section. The dimensions and reinforcement details of the beams are illustrated in Figure 1. The beams used in the test are two batches of beams of different sizes. For the first beam, the width (b) is 120 mm, height (h) is 200 mm, total length (L) is 1500 mm, and the net span (L_0_) is 1200 mm, using HRB400 rebar (Xin Di Yuan Materials Co., LTD., Liaocheng Development Zone, Liaocheng, China). Because the first batch of designed beams is different from the beam structure used in the actual project, its size was improved to make it more close to the actual engineering results and extend its span. For the second beam, the width (b) is 150 mm, height (h) is 200 mm, total length (L) is 2300 mm, and the net span (L_0_) is 2000 mm, also using HRB400 reinforcement. The longitudinal reinforcement in the beam with a total length of 1500 mm consists of two 16 mm-diameter secondary steel bars, and the stirrups are made of primary steel bars with a diameter of 8 mm. In contrast, the beam with a total length of 2300 mm uses three 12 mm diameter threaded rebars for longitudinal reinforcement, two Φ8 mm circular rebars for vertical reinforcement, and six double-legged circular rebars for the stirrups. The spacing between stirrups in the shear span section is 150 mm. The reinforcement layout was designed according to the theory of reinforced concrete structure design, and the reinforcement ratio and spacing were different. Two-point tests were used for both beams. The hydration method determined that the alkali-activated magnesium sulfate cement used in this study contains 62.0% of light-burned magnesia powder (MgO) with an active magnesium oxide (α-MgO) content and 84.77% of heavy-burned magnesia powder (Z-MgO). The median particle sizes (D50) of the two magnesia powders measured using the MicrostracS3500 laser (Verder Shanghai Instruments and Equipment Co., Ltd. in Shanghai, China) particle size analyzer were 46.45 μm and 47.04 μm, respectively. Size distribution curve of magnesium oxide as shown in Figure 2.

#### 2.1.3. Preparing the Specimens

**Figure 3:**
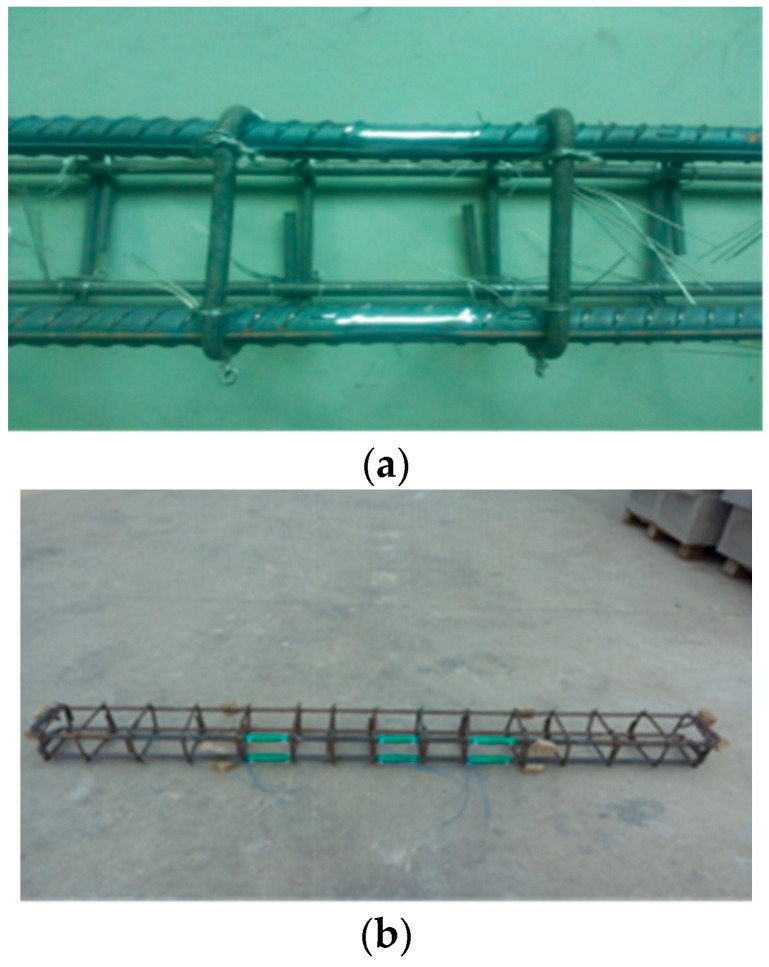

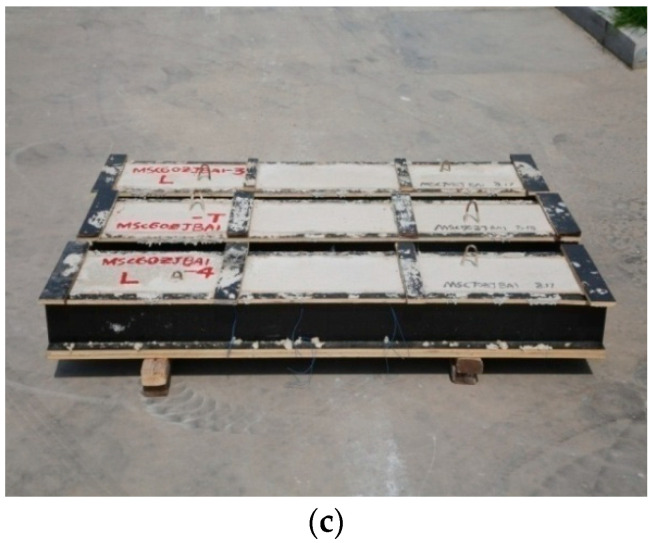
Beam making and maintenance. (**a**) Grinding before attaching strain gauges to beam reinforcement. (**b**) Attaching strain gauges to beam reinforcement. (**c**) Pouring the beam.

After the completion of tying the reinforcement cage, adhesive strain gauges were attached to the hoop bars, stirrups, and longitudinal bars of the corresponding positions. The longitudinal bars and stirrups were then wrapped with plastic sleeves and further enveloped with insulating adhesive tape, with plastic clips used instead of iron wires for binding. Upon the completion of fabricating a reinforcement cage, an ohmmeter was immediately employed to inspect the line insulation of the strain gauges, as shown in Figure 3a,b.

The strain gauges utilized are BX120-3AA rubber-based resistance strain gauges manufactured by Jinli Sensing Element Factory in Xingtai, China. With a grid length of 5 mm and width of 3 mm, the resistance value is (120.0 ± 0.2%) Ω, and the sensitivity coefficient is 2.032 ± 0.30%. To ensure the accuracy of the steel stress data, the quality of the strain gauge attachment was maximized in this experiment. As depicted in Figure 1, the surface of the area where the strain gauges were attached to the reinforcement was first polished smooth. The strain gauges were then adhered using 502 adhesives, with two strain gauges attached to the longitudinal bars at midspan and protective measures against impact and water using 704 silicones. It was essential to ensure that the resistance piece was free from open circuits and damage, as well as insulation between the resistance piece and the specimen, while minimizing the weakening effect on the steel section due to strain gauge attachment.

After the completion of reinforcement cage binding, concrete pads, 25 mm in height, previously prepared according to design requirements, were respectively bound to the bottom and sides of the longitudinal bars. Concrete was cast horizontally using wooden molds, with the specimens cast in two batches. A 500 L forced mixer was used for mixing, and based on the dimensions of the molds and the capacity of the mixer, each batch involves casting one set of beams and three 100 mm × 100 mm × 100 mm test blocks. The concrete was mixed using the method of first generating magnesium sulfate cement mortar and then filling it into the molds of the concrete components, with compaction achieved using a vibrating rod for one-shot forming. After pouring, as depicted in Figure 3c, the specimens were placed indoors and covered with plastic film for curing, with the curing temperature maintained at 20–23 °C and relative humidity at 60%. The three 100 mm × 100 mm × 100 mm test blocks were used to measure the strength of BMSC concrete.

### 2.2. Test Method for Basic Mechanical Properties

**Figure 4:**
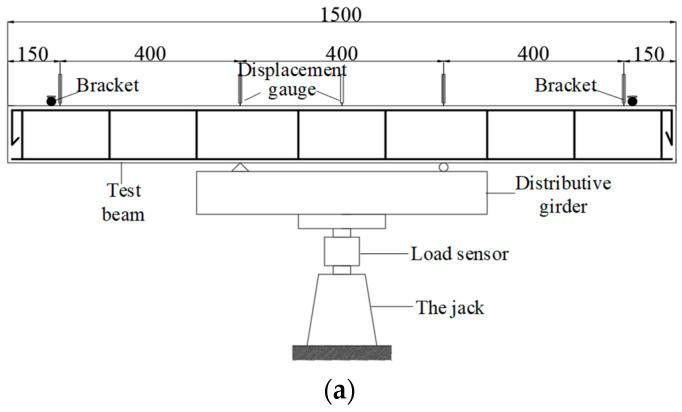

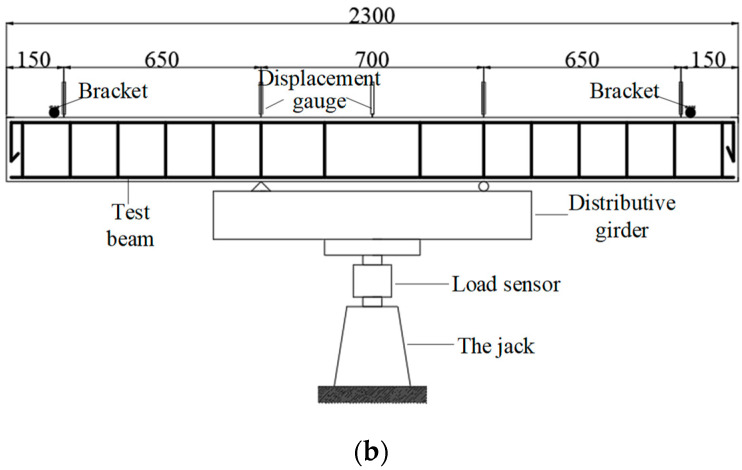
The schematic diagram of the normal section bending loading device. (**a**) L = 1500 mm. (**b**) L = 2300 mm.

The experiment was conducted at the structural laboratory of Nanjing University of Aeronautics and Astronautics in Nanjing, China. Table 4 shows the main parameters of beams subjected to normal section damage. A four-point loading method was used; for instance, for a beam with a length (L) of 2300 mm, the test setup is depicted in Figure 4. During the loading process, a 50-ton load sensor measured load variations. The numbers of steel bar strain gauges, concrete strain gauges, and displacement sensors were recorded using DH3818-2 (Donghua Testing Technology Co., Ltd. in Taizhou, Jiangsu Province, China) static strain gauges. To observe the initiation and progression of cracks and to measure their width, the SW-LW-201 (Shenborui Instrument Co., Ltd. in Shenzhen, China) crack observation instrument was utilized. Five YWC-50 displacement sensors (Beijing Yiyang Strain and Vibration Testing Technology Co., Ltd. in Beijing, China) were installed at specific points on the test beam: both ends, two loading points, and the mid-span position. These sensors measured the settlement displacement at the supports and the deflection at the mid-span to determine the overall deformation of the beam.

To measure the stress in the steel bars during loading, one strain gauge was affixed at the mid-span of the longitudinal bar at the bottom of the beam and near the loading point area, totaling six strain gauges. The sensitive grid size of each steel bar strain gauge was 3 mm × 2 mm. To measure the deformation of the test beam (concrete strain), five strain gauges were arranged along the height of the section in the pure bending area of the beam. This setup was intended to verify whether the section strain of the reinforced concrete beam conforms to the plane section assumption. One strain gauge was attached in both the tension and compression zones to investigate the strain development in these areas when the normal section of the reinforced concrete beam is bent and damaged. The arrangement of strain gauges on the test beam is illustrated in Figure 1. A preloading of 10 kN was applied before the formal loading to ensure proper contact between the components of the loading system and to check the functionality of the instruments. During formal loading, each test beam was loaded in increments of 1 kN per stage before cracking. Once cracking occurred, all cracks were observed, and each stage was loaded with 5 kN. The loading continued in 2 kN increments per stage until the end of the test.

## 3. Experimental Results and Analysis

### 3.1. The Development and Failure Mode of Cracks

**Figure 5:**
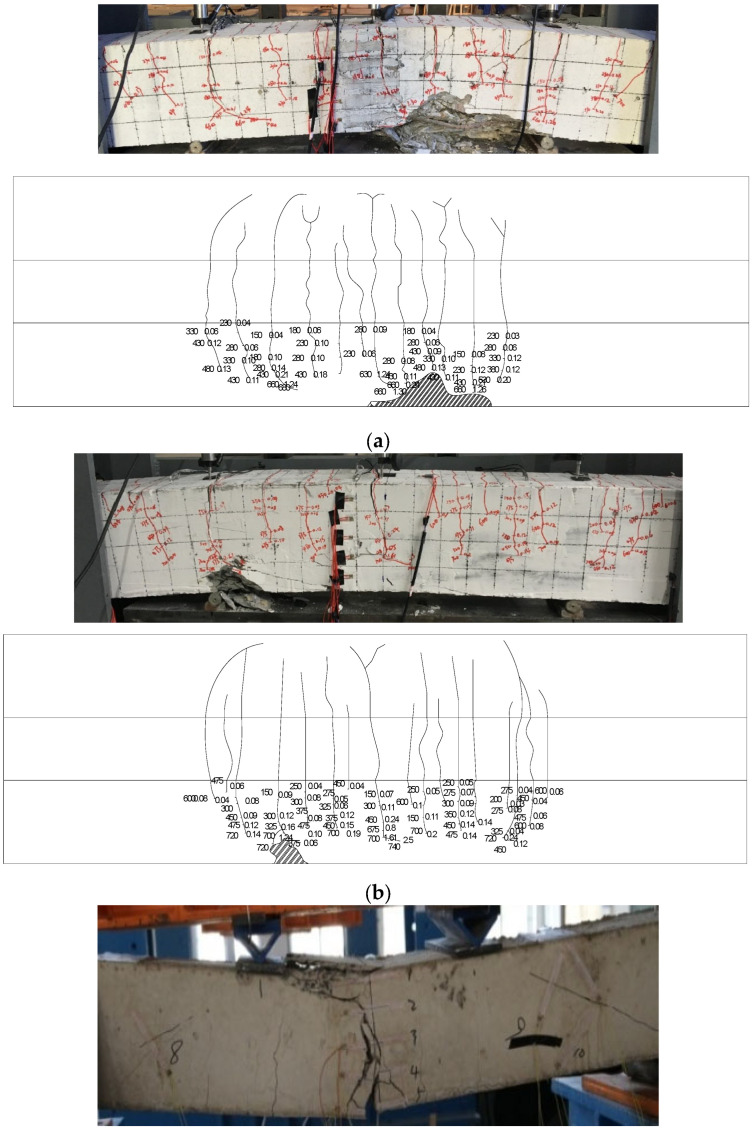

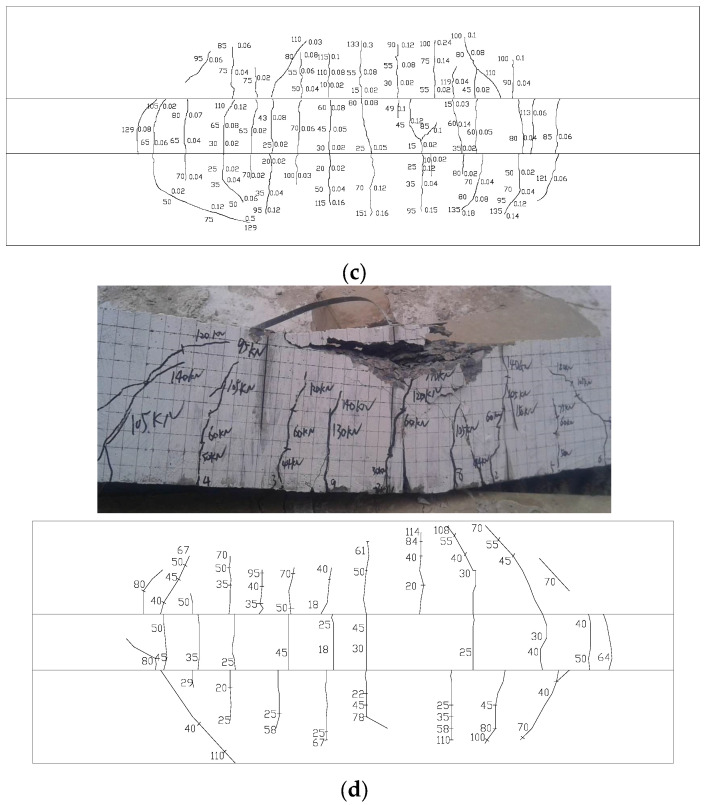
Beam failure mode and three-sided crack expansion diagram. (**a**) PC40; (**b**) JM40; (**c**) JM40a; (**d**) JM50.

The mechanical properties test of the beam revealed that the failure process of the BMSC concrete beam includes distinct stages: elastic, plastic, steel bar yielding, and limit state, leading up to failure. During the initial elastic stage of loading, the stress was proportional to the strain. When the load reached 24% of the ultimate load, cracking occurred at the beam’s mid-span, and the stress in the longitudinal reinforcement increased as the concrete in the tension zone gradually ceased to function. As the load increases, the mid-span cracks expanded, both upwards and in width. Upon reaching 75% of the ultimate load, the number of cracks stabilized, with the maximum crack width being 0.3 mm. Following the yielding of the main reinforcement, there was a sudden increase in strain, rapid deflection of the beam, and widening of the cracks. The beam eventually failed due to the crushing of the concrete in the compression zone. The failure of the beam’s normal section begins with the yielding of the steel bar in the tension zone, followed by the crushing of the concrete in the compression zone, resembling a ductile failure process. The average crack spacing in alkali magnesium concrete beams was significantly smaller than in ordinary concrete beams, with JM40 beams having smaller crack spacing than JM50 beams. Figure 5 illustrates the crack development in the partial normal section flexural performance test beam.

Experimental results indicate that the cracking and ultimate loads for the OPC (PC40) beam were 15 kN and 68 kN, respectively. For the BMSC (JM40) beam, these loads were 15 kN and 69 kN. Comparing the flexural performance of the PC40 and JM40 beams, the JM40 beam demonstrates a significantly higher cracking load, suggesting that under identical section dimensions, material properties, and longitudinal reinforcement ratios, the type of concrete material is the main factor influencing the failure mode of bending beams, with BMSC concrete notably increasing the cracking moment [22].

Figure 5 shows that both the reinforced Portland Cement concrete beam and the reinforced BMSC concrete beam experienced normal section bending failure, with similar crack development and failure modes. Characteristics of the OPC beam included slight noise upon cracking, with cracks mostly concentrated in the mid-span and loading point areas. The first crack typically extended through 30% to 70% of the beam height. As the load increased, cracks expanded along the beam height, with the crack width initially increasing slowly and then rapidly surpassing 1.5 mm. However, the crack length increased slowly in the later stages of loading. When new cracks appeared, there was a 2–5 kN drop in the load-bearing capacity. In contrast, the reinforced BMSC beam, in its early loading stages, primarily developed cracks in the mid-span area, with a higher number of cracks under the same load compared to the reinforced OPC beam. Compared to JM40 beam results, the JM40a beam showed an increase in the number of bending cracks and a decrease in crack height. Meanwhile, the JM50 beam exhibited fewer bending cracks, but these were concentrated in several wider cracks.

### 3.2. Verification of Plane Section Assumption

**Figure 6:**
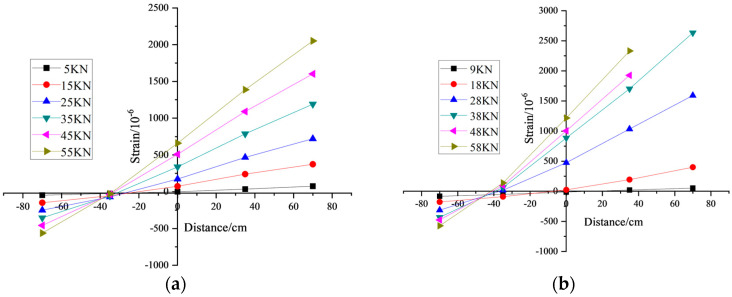
Strain of measuring points at different heights of mid-span section of the beam. (**a**) OPC; (**b**) BMSC.

Figure 6 presents the strain distribution across the mid-span section of both OPC concrete beams and BMSC concrete beams. As observed from Figure 6, from the onset of loading to the point of failure, the average strain distribution along the height of the concrete section was approximately linear. Additionally, the strain experienced by the tensile reinforcement was akin to that of the concrete under equivalent load levels. With increasing load, the neutral axis height gradually shifts upwards. Consequently, this strain distribution pattern across the section adheres to the plane section assumption, indicating effective bonding between the tensile reinforcement and the surrounding concrete.

### 3.3. Load–Deflection Curve of Beam

**Figure 7:**
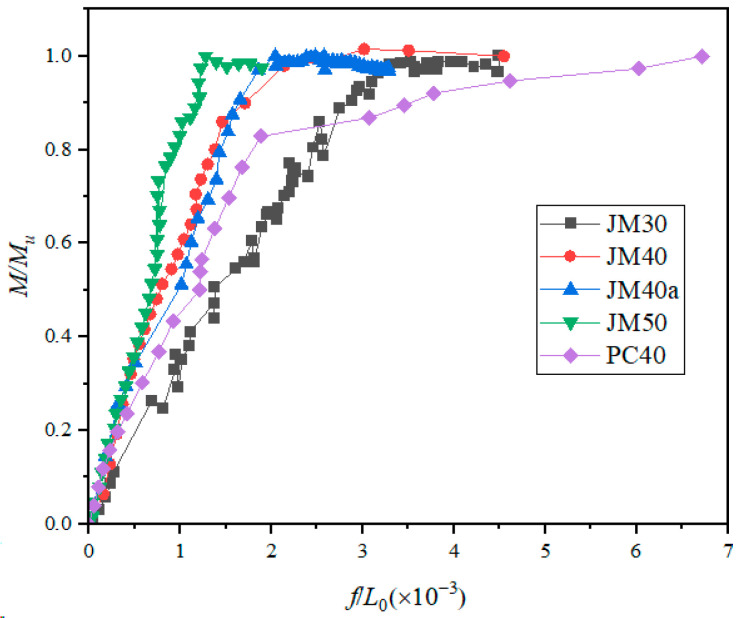
Relative bending distance-deflection curves of Portland Cement concrete beams and BMSC concrete beams.

The actual displacement of the beam is determined by calculating the difference in displacement between the mid-span and the support. The load-relative bending distance-deflection curve for both Portland Cement concrete and BMSC concrete beams under various loads is depicted in Figure 7. This figure illustrates that the relative deflection of the test beam increased with the load’s relative bending distance. When the load bending distance reached the beam’s yield load moment, the beam’s steel bars yield and the beam entered the plastic stage, marked by a sharp increase in deflection leading to beam failure. The relationship between the load bending distance and deflection was linear. However, the curve slope for BMSC concrete beams was less steep than that for OPC beams, indicating that at an equal concrete strength, the stiffness of BMSC concrete beams was greater than that of OPC beams. Under identical loads, the deflection of the BMSC concrete beam was smaller, suggesting that OPC beams had better ductility. A comparison of the bending moment-deflection curves of BMSC concrete beams with different strength grades revealed that the curve slope increases with the strength of the concrete beam. This indicated that increasing the strength of BMSC concrete also increased its stiffness. Consequently, BMSC beams with a higher strength grade were more prone to cracking under the same bending deformation.

**Figure 8:**
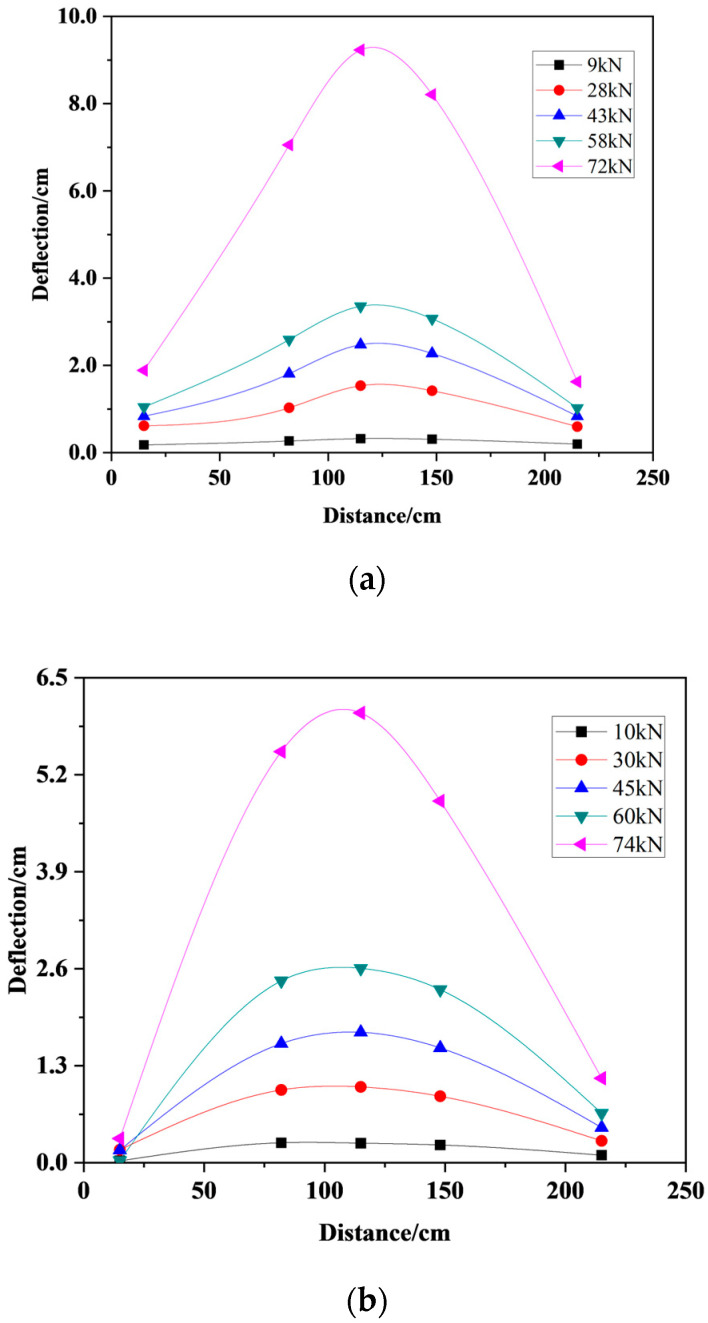
Deflection-length curve of the beam. (**a**) PC40; (**b**) JM40.

Figure 8 shows the deflection-length curve of the beam under different loads. It can be seen from the figure that the deflection distribution of the PC40 beam and JM40 beam along the transverse direction of the steel bar was basically the same. The overall deformation of the JM40 beam was symmetrical. In the loading state, the deflection of the middle position of the beam was the largest, and the deflection near the loading position was the smallest. It can be seen from the Figure 8 that the deflection of the same position of the PC40 beam and JM40 beam was different under the same load, and the PC40 beam would had a larger deflection. Before the load reached the ultimate load, the deflection of the beam increased relatively evenly. When it was close to the ultimate load, the overall deflection of the beam would suddenly increase.

In comparing the experimental results of PC40 and JM40 beams, it was observed that the load-deflection curve of BMSC concrete beams was similar to that of ordinary concrete beams. The load-deflection curve of reinforced BMSC concrete beams could be roughly divided into three stages. The first stage, before beam cracking, spanned from the beginning of loading to the onset of cracking. This phase was the elastic stage. At the initial loading stage, the load on the beam was small, and the section strain was also minor, resulting in a linearly rising load-mid-span deflection curve. The concrete operated elastically, with the load being proportional to the deflection. The characteristics of both BMSC and OPC beams were essentially the same during this phase. When the load reached 12 kN, the concrete strain in the tensile zone of both BMSC and OPC beams attained the ultimate tensile strain of concrete, and small cracks started to form in the tensile zone. The initial central crack width in the ordinary cement beam was 0.04 mm, while it was comparatively smaller in the BMSC beam at 0.02 mm. As the load continued to increase, the concrete in the tensile zone cracked further.

In the second stage, spanning from beam cracking to longitudinal reinforcement yielding, the load increased beyond the cracking load. The first crack appeared in the weaker sections of concrete in the pure bending section or near the loading point, typically extending through 30% to 70% of the beam height. As the load increased, existing cracks gradually widened and extended upwards. When the load reached 63 kN, the central crack width of the OPC beam was 1.24 mm. At 60 kN, the BMSC beam exhibited a penetrating oblique crack at the support, with a central crack width of 0.36 mm.

The third stage was from the yielding of the steel bar to the crushing of the concrete in the compression zone, marking the failure stage. When the load reached 68 kN, the vertical cracks at the center of the OPC beam began to develop horizontally. At 69 kN, the vertical cracks in the center of the BMSC beam followed a similar pattern, and the internal steel bars of the beam yielded. In a specific area on both sides of the main crack at the top of the beam, the concrete in the compression zone underwent significant plastic deformation, forming a concentrated area of plastic deformation. At this point, the stress and strain in the concrete compression zone increased sharply, leading to the crushing of the concrete in this zone and the ultimate destruction of the beam.

### 3.4. Bearing Capacity Analysis

The normal section cracking load of the flexural reinforced concrete beams was determined by observing and analyzing the load-deflection curve. The ultimate bearing capacity was defined as the load at which the concrete’s tensile steel bar yielded. Notably, the maximum mid-span deflection (*f*_max_) refers to the deflection value at the corresponding ultimate bearing capacity, rather than the maximum deflection measured in the test. Through the use of a crack observation instrument, the maximum width of multiple bending cracks was tracked and recorded. Table 5 presents the number of cracks (*N*), the load at the first crack (*P*_cr_), the ultimate load (*P*_u_), and other related test results of the normal section bending test beams.

From the data in Table 5, it is evident that the ultimate load values of the PC40 and JM40 beams were approximately equal. However, the maximum mid-span deflection of JM40 beams was less than that of PC40 beams, with *f*_max_ being 3.8 mm and 2.6 mm, respectively. This suggests that, for beams of the same strength grade, the bending resistance of BMSC beams was stronger than that of OPC beams. Comparing the results of JM30, JM40a, and JM50 beams revealed that for BMSCC beams of the same size, the larger the strength grade, the smaller the *f*_max_. This observation aligns with the conclusions drawn in Section 3.3.

### 3.5. Strain Analysis of Steel Bar

**Figure 9:**
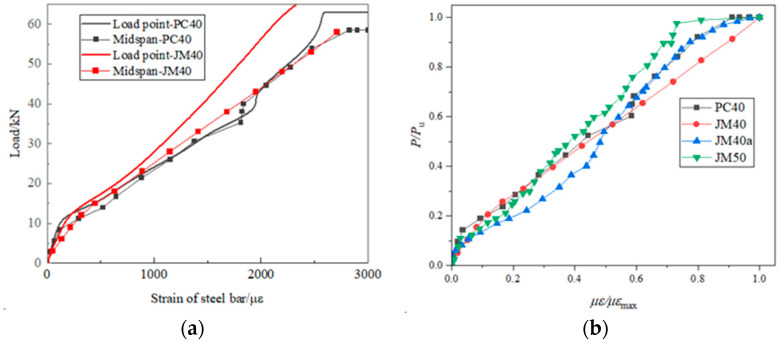
Load-strain curve of steel bar in flexural reinforced concrete beams under longitudinal tension. (**a**) OPC; (**b**) BMSC.

Figure 9 illustrates the load-longitudinal tensile steel strain curve of reinforced concrete beams. Figure 9a presents the stress-strain curve of the steel bar at the mid-span and loading points of both the PC40 and JM40 beams. Meanwhile, Figure 9b displays the stress–strain curve of the steel bar at the mid-span position of the PC40, JM40, JM40a, and JM50 beams. From Figure 9a, it was observed that the steel bars in both OPC and BMSC beam specimens did not enter the yield stage, possibly due to the high yield strength of the HRB400 steel bars. Under a load of 0–10 kN, the OPC beam specimen and its steel bar were in a full cross-section working state, with both the concrete and steel bar undergoing joint stress. At this stage, the strain in the steel bar was minimal and essentially consistent with the component’s deformation. As the load increased, the specimen cracked, causing the concrete in the tensile zone at the crack to cease functioning. The tensile force of the cracked concrete was then borne by the steel bar, leading to an abrupt change in the steel bar’s strain. Figure 9a also revealed that under the same load, the mid-span strain of the BMSC concrete beam was greater than that at the loading point, indicating a more pronounced flexural strain in the mid-span area. This might be related to the crack position in the BMSC beam. In contrast, the steel strain at the loading point of the OPC beam followed a similar development pattern and value as that in the mid-span area. This could be attributed to the more uniform distribution of cracks in the OPC beam, resulting in noticeable bending strain in the steel bars at both the mid-span and loading points. As depicted in Figure 9b, the stress–strain curve of the steel bars at the mid-span position did not show significant changes with varying concrete strength grades and beam sizes. The slope and development pattern of the curve remained essentially consistent.

### 3.6. The Width and Number of Cracks

**Figure 10:**
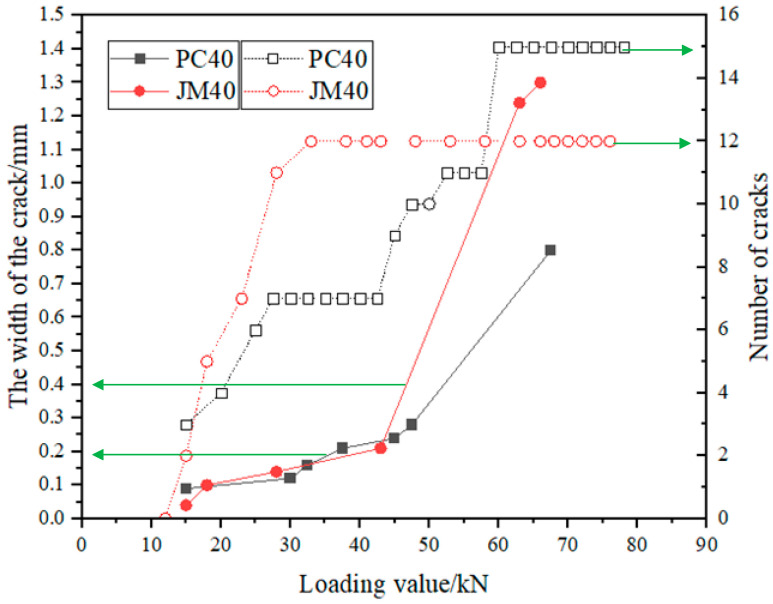
The relationship between the crack width and the number of cracks and the load of reinforced concrete beams.

Figure 10 depicts the relationship among the crack width, the number of cracks, and the load on the reinforced concrete beam specimen. From the data shown in Figure 10, it was observed that cracking in the specimen commenced when the load reached 12 kN. In the load range of 12 to 57 kN, the number of cracks in the BMSC beam was fewer than that in the OPC beam, yet the crack widths were similar. As the load continued to increase, both the number of cracks and the crack width in the specimen significantly increased during the later stages of loading. When the load exceeded 60 kN, the crack width in the OPC beam became noticeably larger than that in the BMSC beam, but the BMSC beam had more cracks than the OPC beam. This indicates that in the later stages of loading, the crack width in the OPC beam was larger and the damage more apparent. Conversely, the BMSC beam, with its numerous but narrower cracks, demonstrated better bending resistance.

## 4. Bending Calculation Model of Beam

It is worth noting that the flexural behavior of ordinary reinforced concrete bending beams mainly depends on the compressive constitutive relation, the bond strength between steel and concrete, and the strength of steel bars [23]. The beams in this test were made of two different materials, both reinforced with the same steel bar. While the crack resistance advantage of the BMSC concrete bending beam could be explained, the flexural bearing capacity advantage was only within a 5% margin of error.

### 4.1. The Relative Boundary Compression Zone Height of the Beam

“Code for design of concrete structures” (GB50010-2010) [24] suggests that the calculation formula of the relative boundary compression zone height (*ξ*_b_) of ordinary reinforced concrete beams is as follows:

(1) $$\xi_{b}=\frac{\beta_{1}}{1+\frac{f_{y}}{\varepsilon_{\mathrm{cu}}^{\prime }E_{s}}}$$

where *β*_1_ is the coefficient of the rectangular stress coefficient diagram, *β*_1_ = 0.8. *f*_y_ is the design value of yield strength of the steel bar. *E*_s_ is the elastic modulus of the steel bar, GPa. $\varepsilon_{\mathrm{cu}}^{\prime }$ is the ultimate compressive strain of concrete, when $\varepsilon_{\mathrm{cu}}^{\prime }$ ≥ 0.0033, $\varepsilon_{\mathrm{cu}}^{\prime }$ = 0.0033. 

Zeng [21] showed that the formula for calculating the height of the relative boundary compression zone of the normal section of the OPC beam is suitable for the BMSC beam. In summary, the measured ultimate compressive strain of the compressive edge of the BMSC concrete beam is $\varepsilon_{\mathrm{cu}}^{\prime }$, *β* = 0.78, and multiple sets of data of the measured value of the *f*_y_ of steel bar were brought into Equation (1). The calculation formula of *ξ*_b_ under flexural failure of the BMSC concrete beams is as follows:

(2) $$\xi_{b}=\frac{\beta }{1+\frac{f_{\mathrm{ys}}}{\varepsilon_{\mathrm{cu}}^{\prime }E_{s}}}$$

### 4.2. Cracking Moment

“Code for design of concrete structures” (GB50010-2010) [24] suggested that the formula for calculating the cracking moment of ordinary reinforced concrete beams is as follows:

*M*_cr_ = γ*f*_tk_*W*_0_ (3)

where, γ is the cross-section resistance moment plastic influence coefficient of BMSC concrete members, and 1.50 is taken for ordinary concrete reinforced beams. *f*_tk_ is the standard value of tensile strength of BMSC concrete, MPa. *W*_0_ is the elastic resistance moment of the cross-section of the BMSC concrete beam, kNm. 

Zeng [16] showed that the calculation formula of the cracking moment of the OPC beam under normal section bending is suitable for the BMSC beam. After systematic analysis, this paper proposes the best formula for the cracking moment *M*_cr_ of the BMSC concrete beam as follows:

*M*_cr_ = *k*_1_γ*f*_tk_*W*_0_ (4)

γ = (0.7 + 120/*h*) γ*_m_* (5)

*f*_tk_ = 0.23 (*f*_cu_)^0.75^ (6)

*I*_0_ = b*y*_0_^3^ + *n*A_s_ (*h*_0_ − *y*_0_)^2^ (7)

*n* = *E*_s_/*E*_c_ (8)

(9) $$E_{c}=\frac{10^{5}}{0.92+\frac{75.6}{f_{cu}}}$$

where, *k*_1_ is the BMSC beam bending cracking moment calculation model correction coefficient, *k*_1_ = 0.8. *M*_cr_ is the crack bend, kN·m. γ_m_ = 1.55. *E*_c_ is the elastic modulus of BMSC concrete [25]. *f*_tk_ is the tensile strength of BMSC concrete [25], MPa. *f*_cu_ is the cube compressive strength, MPa.

The test data are substituted into Equation (4) to obtain the calculation results. The test results of the crack bend of the BMSC concrete beam are compared with the theoretical calculation results, as shown in Table 6. According to the data in the table, the average value of *M*_cr_^t^/*M*_cr_^c^ is 0.986, the standard deviation is 0.098, and the coefficient of variation is 0.099. It can be seen from the table that the fitting effect is good and within a reasonable error range.

### 4.3. Ultimate Bending Moment

“Code for design of concrete structures” (GB50010-2010) [24] suggested that the calculation formula of the ultimate bending moment of ordinary reinforced concrete beams is as follows:

*M*_u_ = ∂_1_*f*_c_*bx* (*h*_0_ − *x*/2) (10)

∂_1_*f*_c_*bx* = *f*_y_A_s_ (11)

where *M*_u_ is the ultimate bending moment, kN·m. ∂_1_ is the coefficient of the rectangular stress diagram. *f*_c_ is the design value of concrete axial compressive strength, MPa. *b* is the width of the rectangular section, mm. *h*_0_ is the effective height of the rectangular section, mm. *x* is the height of concrete compression zone of the equivalent rectangular stress diagram, mm. *A*_s_ is the cross-sectional area of the longitudinal reinforcement in the tensile zone, mm^2^.

Zeng [16] showed that the calculation formula of the ultimate bending moment of the OPC beam under a normal section bending state is suitable for the BMSC beam. In summary, after systematic analysis, this paper suggests that the best formula for the flexural ultimate bending moment of the normal section of the BMSC concrete beam is as follows:

*M*_u_ = *k*_2_∂_1_*f*_c_*bx* (*h*_0_ − *x*/2) (12)

∂_1_*f*_c_*bx* = ∂_2_*f*_y_A_s_ (13)

*f*_c_ = 0.8053*f*_cu_ + 0.4141 (14)

where *k*_2_ is the BMSC beam flexural ultimate bearing capacity calculation model correction coefficient, *k*_2_ = 1.54. *f*_c_ is the design value of concrete axial compressive strength [25], MPa.

The test results are compared with the theoretical calculation results of Equation (10), and the detailed comparison results are shown in Table 7. According to the data in the table, the average value of *M*_u_^t^/*M*_c_^u^ is 0.992, the standard deviation is 0.043, and the coefficient of variation is 0.043. It can be seen from the table that the fitting effect is consistent and within a reasonable error range.

## 5. Conclusions

(1) BMSC beams have similar mechanical properties to OPC beams. The normal section stress process of the BMSC concrete (BMSC) beam was similar to that of the ordinary concrete beam, exhibiting distinct elastic and plastic stages, steel bar yields, and limit states, while conforming to the plane section assumption.
(2) The cracking forms of BMSC beam and OPC beam were different under a load. There was no significant difference between the cracking load values and ultimate load values of ordinary concrete beams and BMSC beams of the same strength grade. The primary differences between BMSC and Portland Cement were evident in the crack width and location. Compared to ordinary concrete beams, the cracks in BMSC were mainly concentrated in the mid-span area and were narrower. In the later stages of loading, the BMSC beams exhibited more cracks than OPC beams, but the cracks were consistently narrower.
(3) The BMSC beam and OPC beam have the same mechanical calculation model. The existing calculation formula for the flexural bearing capacity of ordinary concrete beams was found to be applicable to BMSC beams. Based on the measured compressive strain values of the BMSC in the pure bending section, the compressive stress–strain curve equation for BMSC, and the measured values of tensile steel bar strain, new calculation formulas for stiffness and cracking in BMSC beams were proposed.
(4) Futures and perspectives. Based on this study, further research will be conducted on members made of magnesium sulfate cement concrete. Due to its excellent toughness, the focus will be on analyzing magnesium sulfate cement concrete as a joint component and investigating related materials for bridge joints.

## Figures and Tables

**Table 1:** Physical and mechanical properties of BMSC.

Cement	Normal Consistency/%	Initial Setting Time/min	Final Setting Time/min	Stability	Compressive Strength/MPa			Break Off Strength/MPa		
					3 d	7 d	28 d	3 d	7 d	28 d
P•O42.5	24.1	121	381	Qualification	17.3	33.1	52.9	3.5	4.8	6.5
BSM52.5	19.2	117	278	Qualification	4.2	52.0	56.5	7.5	11.9	13.7

**Table 2:** Chemical components of LBM, FA, and SG.

Constituent/%	Light Burned MgO	Fly Ash	Slag
MgO	87.3	1.31	6.03
SiO_2_	2.87	54.88	28.15
CaO	1.34	4.77	34.54
Fe_2_O_3_	0.39	1.16	0.32
Al_2_O_3_	0.12	26.89	16.02
SO_3_	--	6.49	1.13
LOI	6.56	--	--
IL	--	3.1	2.88

**Table 3:** Concrete mix proportion.

Concrete	Material Components/kg·m^−3^						
	The Strength of Concrete	Cement	Stone	Sand	Water	Slag	Fly Ash
OPC	C40	290	1130	700	140	70	40
BMSC	C30 [21]	530	1131	679	141	——	——
	C40	530	1078	719	143	——	——
	C40 [21]	530	1131	679	158	——	——
	C50 [21]	530	1137	668	132	——	——

**Table 4:** Main parameters of beam with normal section failure.

Beam	Concrete	b × h/mm^2^	Diameter of Main Reinforcement/mm	Reinforcement Ratio	Measured Strength of Concrete/MPa	Standard Deviation/Mpa	Tensile Strength/MPa
JM30	BMSC	120 × 200	Φ14	1.43%	31.4	+1.85	2.73
JM40	BMSC	150 × 200	Φ12	1.93%	45.1	+3.02	3.67
JM40a	BMSC	120 × 200	Φ14	1.43%	45.0	−3.60	3.66
JM50	BMSC	120 × 200	Φ14	1.43%	50.6	+3.49	4.10
PC40	OPC	150 × 200	Φ12	1.93%	41.2	−3.34	1.82

**Table 5:** *M*_cr_ and *M*_u_ test results of normal section flexural reinforced concrete beam specimens.

Beam	*P*_cr_/kN·m	*P*_u_/kN·m	*P*_cr_/*P*_u_	*f*_max_/mm
JM30	18	119	0.15	3.95
JM40	9	69	0.25	2.60
JM40a	12	152	0.08	3.05
JM50	17	118	0.14	0.99
PC40	12	68	0.24	3.80

**Table 6:** Summary of cracking bending distance comparison results of beams.

Cement	Section Size /mm^2^	Diameter of Main Reinforcement /mm	*M*_cr_^t^ /kN·m	*M*_cr_^c^ /kN·m	*M*_cr_^t^/*M*_cr_^c^
JM30	150 × 200	14	2.2	2.3	0.96
JM40	150 × 200	12	3.9	3.4	1.14
JM40a	120 × 200	14	2.5	2.4	1.04
JM50	120 × 200	14	3.2	3.4	0.94
PC40	150 × 200	12	2.9	3.4	0.85

Note: *M*_cr_^t^ is the test value of the cracking moment; *M*_cr_^c^ is the calculation result of the theoretical formula of the cracking moment.

**Table 7:** Summary of cracking moment comparison results of beams.

Cement	Section Size /mm^2^	Diameter of Main Reinforcement /mm	*M*_u_^t^ /kN·m	*M*_u_^c^ /kN·m	*M*_u_^t^/*M*_u_^c^
JM30	120 × 200	14	24.2	23.8	1.02
JM40	150 × 200	12	22.4	22.3	1.01
JM40a	120 × 200	14	24.2	23.6	1.03
JM50	120 × 200	14	22.0	24.2	0.91
PC40	150 × 200	12	22.1	22.3	0.99

Note: *M*_u_^t^ is the ultimate bending moment test value; *M*_u_^c^ is the calculation result of the theoretical formula of the ultimate bending moment.

## Data Availability

Data are contained within the article.
